# Making Visible the Invisible: Automatically Measured Global and Regional Brain Volume Is Associated with Cognitive Impairment and Fatigue in Multiple Sclerosis

**DOI:** 10.3390/bioengineering10010041

**Published:** 2022-12-29

**Authors:** Stefano Ziccardi, Francesca Benedetta Pizzini, Maddalena Guandalini, Agnese Tamanti, Cecilia Cristofori, Massimiliano Calabrese

**Affiliations:** 1 Neurology Section, Department of Neurosciences, Biomedicine and Movement Sciences, University of Verona, 37134 Verona, Italy; 2 Department of Diagnostics and Public Health, University of Verona, 37134 Verona, Italy

**Keywords:** multiple sclerosis, brain volume, MRI analysis, cognitive impairment, fatigue

## Abstract

In multiple sclerosis (MS), the transition from relapsing-remitting to the secondary-progressive phase is characterized by a progression independent of relapse activity (PIRA), resulting in physical disability accumulation and invisible symptoms, i.e., fatigue and cognitive impairment (CI). These symptoms are related to neurodegenerative processes and have been correlated with MRI measures of brain atrophy only at a group level; however, the application in clinical practice of atrophy-based measurements for single-patient evaluation is yet to be fully investigated. In the present study, we aimed to evaluate the association between brain atrophy, measured with easy-to-use automatic software, and the “invisible” MS symptoms of cognition and fatigue. A total of 69 MS patients were included in the study; cognitive impairment and fatigue (FSS) (in addition to neurological disability, EDSS) were assessed and correlated with brain volumes calculated using the automated software QyScore^®^ which is validated for single-patient use in the clinical setting. Results showed that the cognitive status was accurately reflected by measures of atrophy, with a sensitivity of up to 90%. CI patients showed a lower volume compared to cognitively normal patients in the whole brain (*p* = 0.017), gray matter (*p* = 0.042), insula (*p* = 0.035), cerebellum (*p* = 0.008), and limbic lobe (*p* = 0.049). FSS was associated with temporal lobe (r = −0.37, *p* = 0.013) and insular (r = −0.36, *p* = 0.019) volumes. The volumes of the same regions were also associated with EDSS. The global/regional atrophy results, assessed with automatic and easy-to-use software, correlated with cognitive and fatigue symptoms, thus supporting the clinical application in routine patient management.

## 1. Introduction

Multiple sclerosis (MS) is a common disease of the central nervous system that represents the most frequent non-traumatic cause of disability in young adults [1]. At a clinical level, MS has always been classified as a heterogenous disease following a two-staged model [2]: the majority of patients experience an initial phase of transient neurological deficits due to acute inflammation activity and partial or complete remission (relapsing-remitting MS, RRMS), then, in a period variable from one to two decades, patients tend to progressively and irreversibly accumulate disability, entering into the secondary progressive course (SPMS) characterized by neurodegeneration and chronic axonal injury processes [3,4,5].

Nowadays, it is accepted that the conversion from RR to SP conditions occurs gradually; thus, the term “transitional” phase of MS has been proposed [6]. It has been demonstrated that, even in the earliest stages of the RR phase, most patients can experience a steady progression and disability accumulation independent from inflammatory events (progression independent of relapse activity, PIRA) and new MRI lesions [7,8]. Therefore, in clinical practice, it is of fundamental importance to monitor each patient’s disease evolution.

Cognitive impairment and fatigue are two of the most typical diagnostic findings associated with this transition between MS phenotypes and could represent “red flags” for clinicians to detect this progression. Cognitive impairment (CI) is frequent in MS, affecting up to 70% of patients at any time of their disease course [9]. Cognitive dysfunctions can be found from the earliest stages of the disease [10], with a severe impact on the quality of life of patients and their caregivers [11]. Several cognitive domains can be impaired in MS, i.e., memory, attention, information processing speed, and executive functioning [12].

Fatigue is also frequently described and represents a disabling symptom for most MS patients, affecting up to 95% of them during their disease course [13,14]. However, MS fatigue is a heterogeneous phenomenon, including not only physical but also mental and cognitive fatigue [15]. Fatigue is reported as the most severe MS-related symptom [16] and as the major contributor to poor health perception and low quality of life [17].

Both CI and fatigue are classified as “invisible symptoms” of MS due to the difficulty in evaluating them in clinical practice [18], considering the lack of time of healthcare professionals during the clinical evaluation and the absence of unique objective and reliable assessment instruments able to detect the real status of patients. Nevertheless, the presence of these symptoms reflects cerebral abnormalities; it has been demonstrated that the association between brain atrophy and both CI [19,20,21,22] and fatigue [23,24,25,26,27] is the strongest correlation among measures of brain damage [15]. However, the quantification of atrophy is not straightforward, and its evaluation in MS patients is limited to research settings or cases with severe clinical deficits. Current software for calculating brain atrophy (FreeSurfer, SienaX, etc.) are mainly used for group analysis since they cannot provide clinical information regarding the brain volumes of single subjects with reference to a normative sample. Therefore, monitoring PIRA patients characterized by a steady progression toward the SP phase remains an unmet need in clinical practice.

In the present study, we tested the possibility of utilizing an automatic FDA/CE-approved software for evaluating and quantifying brain atrophy (QyScore^®^) that could be easily performed in MS clinical practice and also employed at a clinical level for single-patient assessments. We aimed to investigate the association between atrophy and the clinically invisible symptoms of cognitive alterations and fatigue.

## 2. Materials and Methods

We enrolled in the present study a total of 69 MS patients (46 females, mean ± SD age = 38.2 ± 12.2 years). All patients underwent a 3T MRI and a neurological evaluation, including the evaluation of clinical disability through the Expanded Disability Status Scale (EDSS) [28]. In addition, a subgroup of 56 patients underwent a comprehensive neuropsychological assessment, 43 of whom completed a fatigue evaluation.

Exclusion criteria were the presence of any concomitant neurological condition (other than MS), substance abuse, hearing impairment, or upper-limb impairment that could interfere with neuropsychological performance.

All participants were recruited at the MS Center of the Verona University Hospital (Verona, Italy). The local Ethics Committee approved the present study. Informed consent was collected from all participants.

### 2.1. MRI Acquisition and Analysis

All images were acquired using a 3T scanner (Achieva, Philips Medical Systems, Eindhoven, The Netherlands) with an 8-channel head coil. The following sets of images were acquired:

- 3D T1-weighted turbo field echo, repetition time (TR) = 8.1 ms, echo time (TE) = 3.7 ms, 180 slices, voxel size = 1 × 1 × 1 mm^3^;
- 3D fluid-attenuated inversion recovery (FLAIR), TR = 8000 ms, TE = 290 ms, inversion time (TI) = 2360 ms, 180 slices, FOV = 1 × 1 × 1 mm^3^.

We used the software QyScore^®^, developed by Qynapse (https://qynapse.com/qyscore, accessed on 28 November 2022), to calculate cerebral volumes by considering 3D T1 and 3D FLAIR images. QyScore^®^ reports generally provide measurements of the global and regional atrophy expressed either in volume (mL), possibly normalized to the intracranial cavity volume (%ICV), or in z-scores and percentiles resulting from the comparison with a wide database of healthy controls. For the present study, we considered whole brain (WB) percentiles and normalized volumes of both global measures, such as the whole brain (WB), grey matter (GM), and white matter (WM), and regional measures, such as the frontal lobe, temporal lobe, parietal lobe, occipital lobe, limbic lobe, amygdala, hippocampus, insula, and cerebellum volumes.

### 2.2. Neuropsychological Assessment

MS patients underwent a comprehensive neuropsychological assessment by using the Brief Repeatable Battery of Neuropsychological Tests (BRB-NT) [29] and the Stroop Test (ST) [30]. The BRB-NT is composed of tests of verbal learning and memory (Selective Reminding Test, SRT); visuospatial learning and memory (10/36 Spatial Recall Test, SPART); visual information processing speed and attention (Symbol Digit Modalities Test, SDMT); auditory information processing speed, calculation, and attention (Paced Auditory Serial Addition Task, PASAT); and semantic verbal fluency within two categories (Word List Generation, WLG). The ST is a test of automatic response inhibition and attention (ST-EIT and ST-EIE).

Moreover, raw scores were adjusted for age, education, and gender: adjusted scores below the cut-off (5th percentile) of the Italian normative data of each test were considered as failed. Patients were clinically classified based on their cognitive performance by using a conservative approach [31]: cognitively normal (CN, no failed subtests) and cognitively impaired (CI). Furthermore, CI patients were specifically subdivided into mild CI (mCI, one or two failed subtests) and severe CI (sCI, at least three failed subtests).

### 2.3. Fatigue

Fatigue was evaluated using the Fatigue Severity Scale (FSS) [32], one of the most used scales to assess subjective fatigue. FSS is a Likert scale composed of nine items, each ranging from 1 (strongly disagree) to 7 (strongly agree): the higher the global score, the higher the subjective fatigue perceived.

### 2.4. Statistical Analysis

SPSS statistic software (SPSS Inc., Chicago, IL, USA, version 24) and the JASP software (JASP Team 2019, version 0.16.1) were used to perform statistical analyses, and design figures and graphs.

Pearson correlation analyses were carried out between brain volume measurements and physical disability (EDSS) and fatigue (FSS).

A Chi-square analysis was performed to compare the prevalence of patients with different percentiles of whole brain volume and cognitive status.

*T*-test analyses were conducted to compare MRI metrics between patients with and without cognitive impairment (CN vs. CI), and ANOVA analyses with post-hoc tests were conducted to compare MRI metrics between patients classified considering a more specific subdivision of the cognitively impaired status (CN vs. mCI vs. sCI).

Results were presented as the means ± standard deviations (SDs) for continuous variables or as medians and interquartile ranges (IQR) for discrete variables. A *p*-value less than 0.05 was considered statistically significant.

## 3. Results

### 3.1. Physical Disability

In total, 14 clinically isolated syndromes (CIS), 50 relapsing-remitting MS (RRMS), and 5 primary progressive MS (PPMS) were enrolled. The sample median [IQR] EDSS was 2.0 [2.0].

**Figure 1:**
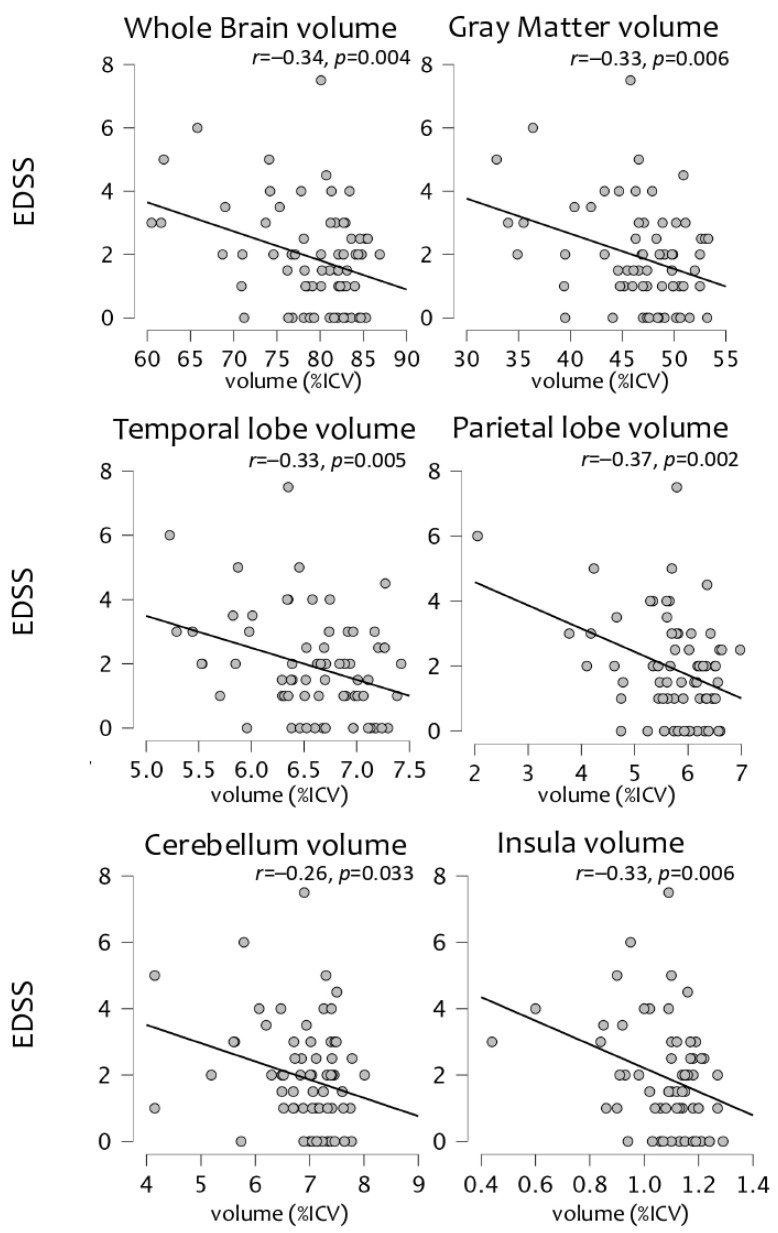
The significant association between physical disability (EDSS) and percentages of intracranial volumes (%ICV), both at a global level (whole brain, gray matter) and in specific regions (temporal lobe, parietal lobe, cerebellum, and insula).

A significant correlation was found between EDSS and the whole brain (r = −0.34, *p* = 0.004) and the grey matter (r = −0.33, *p* = 0.006) volumes normalized to the ICV, as well as the regional normalized volumes of the parietal lobe (r = −0.37, *p* = 0.002), temporal lobe (r = −0.33, *p* = 0.005), cerebellum (r = −0.26, *p* = 0.33), and insula (r = −0.33, *p* = 0.006) (Figure 1).

### 3.2. Fatigue

**Figure 2:**
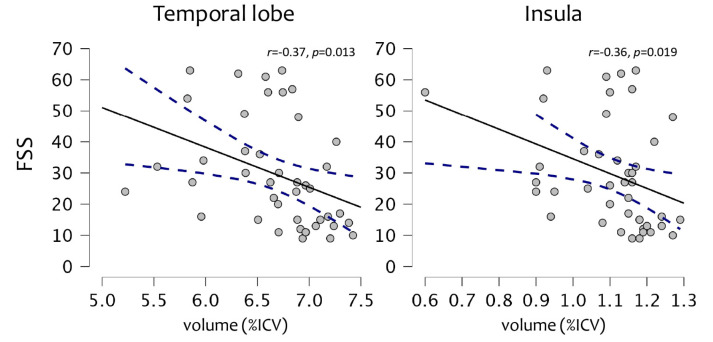
A significant association between fatigue (FSS) and percentages of intracranial volumes (%ICV) in the temporal lobe and the insula.

The mean FSS score of the sample was 29.5 ± 17.4. A significant correlation was observed between FSS and the volumes of the temporal lobe (r = −0.37, *p* = 0.013) and insula (r = −0.36, *p* = 0.019) (Figure 2).

### 3.3. Cognitive Status

The sample was composed of 26 CN patients and 30 CI patients (20 mCI and 10 sCI).

The concordance between the prevalence of patients classified using percentiles of whole brain volume and cognitive status was analyzed. A Chi-square analysis showed a significant result (*p* = 0.043), suggesting the association between the two methods of classification.

A valuable concordance was found between these two methods, in particular for patients with lower cognitive functioning (<50 percentile): 24 out of 30 patients showed a reduction in brain volume (<50 percentile) with a sensitivity of 80%. Moreover, focusing on patients with the lowest cognitive functioning (<25 percentile), almost all of them (9 out of 10) showed a decrease in brain volume (<50 percentile) with a sensitivity of 90%. However, only 6 out of 24 patients with a higher cognitive functioning (>50 percentile) showed a higher brain volume (>50 percentile) with a specificity of 35%. Results are shown in Table 1.

**Figure 3:**
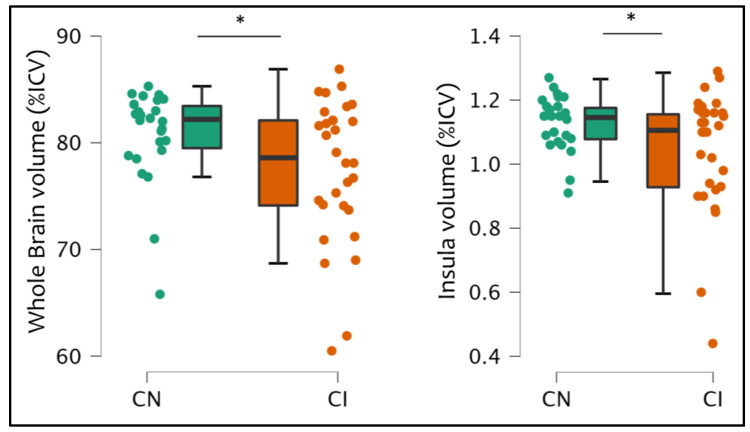
The significant differences among cognitive groups of patients (cognitively normal CN vs. cognitively impaired CI) regarding percentages of intracranial volumes (%ICV) of both the whole brain and the insula. *: *p* < 0.05.

Patients with CI showed a significant decrease in whole brain volume (*p* = 0.032) and insular volume (*p* = 0.035) compared to CN (Figure 3).

**Figure 4:**
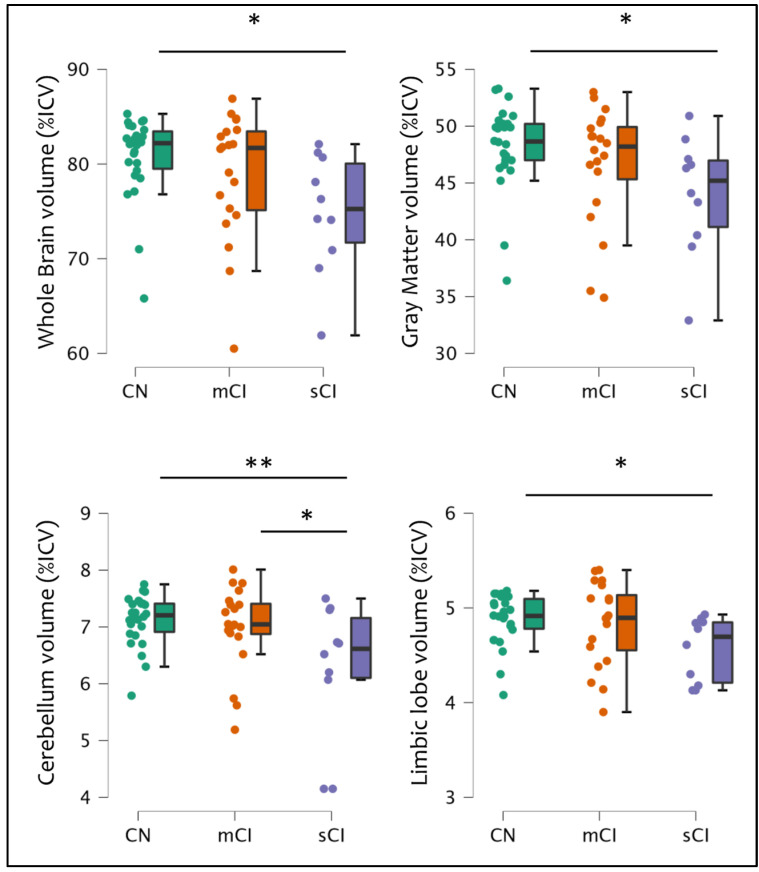
The significant differences among cognitive groups of patients (cognitively normal CN vs. mild cognitively impaired mCI vs. severe cognitively impaired sCI) regarding the percentages of intracranial volumes (%ICV) both at a global level (whole brain, grey matter) and in specific regions (cerebellum, limbic lobe). *: *p* < 0.05; **: *p* < 0.01.

Specifically, patients with a severe cognitive impairment showed a decrease in whole brain volume (CN vs. sCI: *p* = 0.032), grey matter volume (CN vs. sCI: *p* = 0.042), cerebellar volume (CN vs. sCI: *p* = 0.008; mCI vs. sCI: *p* = 0.042), and limbic lobe volume (CN vs. sCI: *p* = 0.049) compared to other patients (Figure 4).

## 4. Discussion

Recent observations have been provided on the evidence that the transition between the relapsing-remitting to the secondary-progressive course of MS is characterized by a gradual conversion mostly characterized by a progression independent of new clinical attacks or new MRI lesions [6,7,8]. Therefore, adequate monitoring should be mandatory to identify such progression promptly. A potential target for monitoring the progression of the disease could be brain atrophy, which reflects the neurodegeneration process leading to the accumulation of MS symptomatology. So far, the efficacious measurement of brain atrophy has been an unmet need for clinicians due to the time-consuming procedure for processing and evaluating neuroradiological images in clinical practice. In the present study, we tested the applicability in the MS clinical routine of QyScore^®^, an automatic tool for the rapid measurement of brain atrophy. QyScore^®^ provides clinicians with specific data on global and regional brain volumes to evaluate brain atrophy and monitor it over time following an automatic FDA/CE-approved process showing a performance comparable to the manual segmentation of expert neuroradiologists [33]. Moreover, Qyscore^®^ provides invaluable details comparing volumes to a reference sample of healthy subjects, to obtain clinical information for evaluating and monitoring brain atrophy on single-patient use.

Our results showed that the brain volume automatically measured with QyScore^®^ is associated with MS clinical symptoms: physical disability, fatigue, and cognitive alterations. Firstly, we found that atrophy in the whole brain, GM, temporal lobe, parietal lobe, cerebellum, and insula was associated with physical disability measured using the EDSS. Physical disability is undoubtedly the most investigated clinical symptom in MS, thanks to the administration of the EDSS that allows the evaluation of several physical domains. Our results corroborated its association with brain atrophy, as we found a significant correlation between EDSS and brain volumes, in line with previous studies [34,35,36].

However, in addition to physical disability, other symptoms may be experienced by patients with MS, in particular cognitive impairment and fatigue. In the present study, we found that higher fatigue levels were associated with higher atrophy in the temporal lobe and insula, while patients who showed cognitive alterations were also those characterized by higher atrophy in key regions for cognitive performances, such as the limbic lobe, the cerebellum, and the insula, as well as in the whole brain and the GM, compared to cognitively normal patients. These results are in accordance with the literature for both cognitive alterations [19,37,38] and fatigue [23,24,25,26,27].

Despite being extremely frequent in MS patients and severely impactful, cognitive impairment and fatigue are too often neglected in clinical practice and have been defined as the “invisible symptoms” of MS [18]. Nevertheless, it is evident that the impact of these symptoms, which often arise together with emotional disturbances of anxiety and depression, on the daily life of patients necessitates the need to increase clinicians’ mindfulness of them [15]. Recent studies are moving in this direction, suggesting incorporating neuropsychological tests and fatigue questionnaires within the EDSS to improve its accuracy as a complete measure of the clinical status of MS patients [39,40]. Moreover, some MS patients show cognitive alterations and fatigue despite a minimum grade of physical difficulties, thus suggesting how these symptoms should be targeted in primary care [41,42,43].

It is important to point out the valuable concordance that we found in this study between the whole brain atrophy and cognitive status, specifically the high sensitivity of Qyscore^®^ in the identification of cognitively impaired patients (80% for both mildly and severely impaired, reaching 90% for only severely impaired). On the other hand, the specificity of Qyscore^®^ in the identification of cognitively normal patients was low (35%), probably due to the great role played by compensation mechanisms (brain and cognitive reserve above all) and by the relatively low ability of a paper-and-pencil test to identify slight cognitive alterations, compared to computerized assessment methods [43]. These results suggest the use of Qyscore^®^ as a screening test at a patient level, able to identify those at major risk of cognitive alterations that should be clinically assessed. From our results emerged the importance of measuring the neurodegenerative processes and monitoring the “silent” progression of those less visible and measurable symptoms caused by MS.

The present study is not without limitations. Firstly, the number of patients included in the analyses is limited and heterogeneous in terms of the number of patients that performed clinical assessments: future studies should increase the sample size and include patients that underwent all the clinical evaluations. Moreover, the current study followed a cross-sectional design: future studies should also consider a longitudinal evaluation of the outcomes measured in the present work, including both the neuroradiological evolution over time and the monitoring of physical/cognitive disabilities and fatigue, to prove the reliability of this MRI tool in MS routine practice. Furthermore, evidence has been provided on the importance of the developmental origins of this health and disease conceptual model regarding the relevance of the prenatal period for later well-being [44,45]: future research should also consider this important perspective in multiple sclerosis as well as in other chronic/neurodegenerative disorders.

## 5. Conclusions

Our results showed that the evaluation of global and regional brain atrophy in clinical practice is feasible and could be implemented in routine clinical practice as a screening test to evaluate the progression of multiple sclerosis disease and to prevent the invisible clinical symptoms of cognitive impairment and fatigue.

## Figures and Tables

**Table 1:** Concordance between the number of patients clustered considering percentiles of whole brain volume and global cognitive functioning. CN: cognitively normal, mCI: mild cognitively impaired, sCI: severe cognitively impaired.

		Whole Brain Volume (Percentiles)			
		0–25	25–50	50–75	75–100
**Cognitive Status**	sCI	8	1	0	1
	mCI	9	6	3	2
	CN	6	11	7	2

## Data Availability

The data supporting the findings of this study are available from the corresponding authors upon reasonable request.
